# Drivers and Barriers to Implementing the Internet of Things in the Health Care Supply Chain: Mixed Methods Multicase Study

**DOI:** 10.2196/48730

**Published:** 2023-09-20

**Authors:** Tjitske J. Heeres, Tri Mikael Tran, Bart A.C. Noort

**Affiliations:** 1 Department of Operations Faculty of Economics and Business University of Groningen Groningen Netherlands

**Keywords:** digital health, drivers and barriers, healthcare logistics, healthcare supply chain, implementation, Internet of Things, supply chain management

## Abstract

**Background:**

Over the past 2 years, the COVID-19 pandemic has placed enormous pressure on the health care industry. There has been an increase in demand and, at the same time, a shortage of supplies. This has shown that supply chain management in the health care industry cannot be taken for granted. Furthermore, the health care industry is also facing other major challenges, such as the current labor market shortage. In the literature, the Internet of Things (IoT) is highlighted as an effective tool to build a more resilient and efficient supply chain that can manage these challenges. Although using IoT in supply chain management has been extensively examined in other types of supply chains, its use in the health care supply chain has largely been overlooked. Given that the health care supply chain, compared to others, is more complex and is under growing pressure, a more in-depth understanding of the opportunities brought by IoT is necessary.

**Objective:**

This study aims to address this research gap by identifying and ranking the drivers of and barriers to implementing IoT in the health care supply chain.

**Methods:**

We conducted a 2-stage study. In the first, exploratory stage, a total of 12 semistructured interviews were conducted to identify drivers and barriers. In the second, confirmatory stage, a total of 26 health care supply chain professionals were asked in a survey to rank the drivers and barriers.

**Results:**

The results show that there are multiple financial, operational, strategy-related, and supply chain-related drivers for implementing IoT. Similarly, there are various financial, strategy-related, supply chain-related, technology-related, and user-related barriers. The findings also show that supply chain-related drivers (eg, increased transparency, traceability, and collaboration with suppliers) are the strongest drivers, while financial barriers (eg, high implementation costs and difficulties in building a business case) are the biggest barriers to overcome.

**Conclusions:**

The findings of this study add to the limited literature regarding IoT in the health care supply chain by empirically identifying the most important drivers and barriers to IoT implementation. The ranking of drivers and barriers provides guidance for practitioners and health care provider leaders intending to implement IoT in the health care supply chain.

## Introduction

### Background and Motivation

COVID-19 exemplifies the sort of disruption by which the health care industry is severely affected. The demand for care increased as an increasing number of people were hospitalized [1,2]. At the same time, shortages of supplies, such as protective equipment and ventilators, occurred due to supply chain disruptions [3,4]. The increased demand for hospital care and supply chain disruptions put even more strain on health care organizations and their personnel, while also dealing with high rates of employee absenteeism due to illness or quarantine obligations [5,6]. Another topical issue that the health care industry is having to deal with is rapidly growing labor market shortages, partly due to the aging population [5,7,8]. Therefore, there is a need to find solutions to be able to better manage disruptions in the future and create a more efficient supply chain. Given the tight health care budgets and labor market shortages in the health care systems of many countries, supporting health care delivery processes through technology is seen as pivotal [9].

One of the relevant supporting technologies for health care is the Internet of Things (IoT), which can be viewed as a connected network of physical objects that sense, monitor, and interact with each other within a company, as well as between companies in a supply chain, to foster effective supply chain management [10]. As IoT creates the possibility to collect, process, and analyze large quantities of real-time data [11], it enables rapid and efficient information sharing [12-14], the creation of a more flexible supply chain [15], and increased levels of visibility throughout the supply chain, thereby strengthening the ability to deal with disruptions [10,16-19]. Consequently, IoT can aid in building a more resilient supply chain [20-23]. For instance, IoT can be deployed to establish links with lower-tier vendors or to automate inventory management, resulting in greater responsiveness and better capacity utilization [10,24]. Even though supply chain management is often seen as a supporting process in the health care industry, logistics and supply chain management are responsible for a significant proportion of health care delivery costs [25,26]. Therefore, the application of IoT is an important opportunity for health care providers.

While other industries are already reaping the benefits of digital technologies, the health care industry has been the slowest to adopt modern information technologies, such as IoT [27]. To date, most IoT research in a health care setting has focused on patient care activities [28]. For instance, IoT is used to collect patient and staff data, to remotely monitor patients’ health indicators, and in emergency warning systems [27]. Even though supply chain management offers a great opportunity to save costs and build a more effective supply chain, it is still regularly seen as a supporting process rather than a core process in the health care industry [29]. Consequently, despite numerous studies on using IoT in supply chain management in other fields, there is a lack of studies investigating the implementation of IoT in health care supply chains. Furthermore, most studies that have been conducted regarding the challenges of implementing digital technologies in health care systems have focused mainly on technical aspects, with social aspects being underinvestigated [30,31]. The current knowledge of IoT applications in supply chain management cannot be directly applied in a health care context because various additional complexities play a role. Sources of these additional complexities include the rather unpredictable intake of patients and the inability to completely forecast a patient’s treatment and need [32], the large number of stakeholders involved in care delivery [27], and the large number of departments and distribution channels [33]. Currently, there is a lack of a full understanding of how these complexities translate into possible barriers to implementing IoT in health care. Also, the key drivers that would push health care staff toward IoT development remain largely unknown.

### Research Objective

Further research as to why IoT is not more widely used in supply chain management within the health care industry could be of great value and address the research gap that has been identified by multiple authors [10,21,31,34]. IoT has the potential to help build resilience in the health care supply chain in multiple ways once the barriers have been addressed. Our research question is thus formulated as follows: “What are the drivers and barriers to implementing the Internet of Things in healthcare supply chains?”

This study contributes to the existing literature (see Multimedia Appendix 1) by addressing the oversight of IoT in health care supply chain management. Additionally, the results of this study will provide practitioners with an overview of potential drivers and barriers they may face when incorporating IoT in the health care supply chain.

## Methods

### Research Design

This mixed methods study aims to identify the drivers and barriers to implementing IoT technology in the health care supply chain. Previous studies have indicated the need for further empirical research to examine the use of IoT and how this could benefit the supply chain [21,34]. Given that this study aims to examine in depth the reasons for the absence of IoT in a real-life context, exploratory research is appropriate, and a cross-sectional case study with interviews is considered the best approach [35]. In order to increase the generalizability and robustness of this study’s findings, multiple health care facilities and logistics organizations were included [35]. Subsequent to the interviews, a survey, which is seen as an appropriate way to establish priorities in a set of elements [36], was conducted in order to determine the relative importance of all the previously identified drivers and barriers.

### Case Selection

In order to increase the external validity of the study [37], a diverse set of cases was selected based on literal and theoretical replication [35]. Potentially relevant cases were identified and approached by email based on purposeful, convenience, and snowball sampling. In total, 17 potential interviewees were contacted, of whom 12 agreed to participate in this study. Those who were not willing to participate were either (1) unavailable due to schedule conflicts (n=2) or (2) did not have sufficient knowledge on the topic (n=3) and referred the researchers to a more appropriate interviewee. This number of interviews proved to be sufficient, as the data analysis showed that saturation was achieved after 7 interviews. An overview of the interviewees and cases can be found in Table 1. For the ranking survey following the interviews, potential respondents were again approached based on purposeful, convenient, and snowball sampling. The interviewees were asked to fill out the survey and distribute it to colleagues involved in supply chain management within their organization. As a result, there was a partial overlap between the interviewees and the respondents. To improve external validity, the survey was distributed to senior managers of large medical equipment suppliers, thereby obtaining input from persons outside of the case companies. As the survey was fully anonymous, its results could not be linked to the interviewees. All the interviewees and survey respondents were Dutch, and the interviews and survey were therefore conducted in Dutch.

**Table 1:** Overview of interviews conducted.

Interviewee	Function	Organization	Size of organization	Duration (minutes)
I1	Account manager	Logistics provider	Four distribution centers the Netherlands	44
I2	Clinical computer scientist	University medical center	Around 12,000 employees	30
I3	Supply chain manager	University medical center	Around 12,000 employees	42
I4	Business process specialist	Organization offering smart solutions	Cooperate with around 38 health care facilities	73
I5	Program manager	University medical center	Around 12,000 employees	45
I6	Contract and supplier manager	General hospital	Around 3000 employees	51
I7	Program manager	General hospital	Around 4500 employees	53
I8	Head of logistics center	Logistics provider	Provide logistics for two hospitals	34
I9	Clinical computer scientist	General hospital	Around 3000 employees	41
I10	Program manager	General hospital	Around 4500 employees	45
I11	Lead enterprise architect	University medical center	Around 12,000 employees	55
I12	Director of marketing, commerce, and innovation	Organization offering smart solutions	Cooperate with around 38 health care facilities	50

### Data Collection and Measurement

#### Exploratory Stage: Semistructured Interviews

Data collection took place between April 2022 and January 2023. The primary sources of data were 12 semistructured interviews that were held in person or through a one-on-one video call. Interviewing is seen as an excellent method for gathering information about opinions, experiences, and complex motivations [38]. Here, open questions were used to create room for flexibility, and probing questions were asked to gain greater in-depth knowledge about interesting topics that were raised by the interviewees [38,39]. The interview guide was designed based on Walker et al [40] with adjustments to reflect the diffusion of innovation theory [41], the technology acceptance model (eg, Davis [42] and Davis et al [43]), and the technology-organization-environment (TOE) framework (Tornatzky et al [44]). The interview guide was jointly developed by the first and second authors (TJH and TMT) and was continuously reevaluated by these authors. The full guide can be found in Multimedia Appendix 2. The interviews were conducted by the first author, TJH (a junior researcher, female, with master’s-level training in qualitative research). Background information about the study and the researcher’s interest in the topic, as well as exploratory questions regarding the interviewee’s knowledge about IoT and the extent to which IoT was being used within the interviewee’s organization, were sent through email in advance to better prepare for the interviews. At the end of the interviews, interviewees were asked if they wanted to discuss anything else. This was done to facilitate the continuous improvement of the interview guide. With the permission of the interviewees, all the interviews were digitally recorded to facilitate their transcription. The interviews lasted between 30 and 73 minutes, as indicated in Table 1. The COREQ (Consolidated Criteria for Reporting Qualitative Research) Checklist was used to warrant the quality of our qualitative research approach [45] (Multimedia Appendix 3).

#### Confirmatory Stage: Ranking With a Survey

In order to validate the importance of the drivers and barriers identified during the interviews, a survey was conducted among participants in the health care supply chain in the Netherlands. Here, respondents were asked to rank the importance of each element, similar to what Baharmand et al [46] did with blockchain technology. The survey design was based on the findings from the exploratory stage of the study; thus, the validity of the survey is ensured through the validity of data analysis in the exploratory stage. A total of 2 ranking methods (selecting the top 5 drivers or barriers and rating each driver or barrier on a 5-point Likert scale) were used to ensure the reliability of the answers. Furthermore, open-ended questions were also asked to confirm the exhaustiveness (ie, validity) of the findings from the exploratory stage. The full survey can be found in Multimedia Appendix 4. In the pilot stage, the survey was tested and previewed. Minor technical and translation-related adjustments were made. The survey was then completed by 26 respondents, who were mainly employed at health care organizations, logistics providers, or suppliers of medical supplies with an average experience of more than 10 years. The IoT knowledge of the respondents and the use of IoT within their organizations ranged from very limited to extensive. The data collection for the confirmatory stage took place from January 2023 to February 2023. A complete overview of the descriptive statistics can be found in Multimedia Appendix 5.

### Data Analysis

#### Exploratory Stage

Following Voss et al [37], the primary data resulting from the interviews were documented, coded, and then analyzed. First, all the interviews were transcribed and documented as soon as possible after each interview. To increase the accuracy of the documentation, follow-up emails were sent to the interviewee to clarify any doubts, and a summary was provided to the interviewee to be checked for any misunderstandings. Subsequently, the data were theoretically coded using ATLAS.ti (ATLAS.ti Scientific Software Development GmbH). Following Strauss and Corbin [47], this involved 3 steps: open coding, axial coding, and selective coding. The first author (TJH) had the lead in the coding process and regularly discussed the analysis with the coauthors (TMT and BACN). This resulted in 15 drivers and 11 barriers to implementing IoT, which were grouped into several aggregate dimensions based on the dimensions identified by Sodhi et al. [48]. In addition, the interviewees were grouped based on their functions. Three clusters emerged (Textbox 1): (1) interviewees who were mainly executing projects (I1, I4, I5, I7, I10, and I12); (2) interviewees working in IT (I2, I6, I9, and I11); and (3) interviewees who were heads of logistics or supply chain managers (I3 and I8). Based on this, within-cluster and cross-cluster analyses were performed as proposed by Eisenhardt [49]. The results of this analysis are discussed in the following section.

**Summary of the clusters.**

**Cluster 1 (interviewees mainly executing projects)**

- I1: Account manager
- I4: Business process specialist
- I5: Program manager
- I7: Program manager
- I10: Program manager
- I12: Director of marketing, commerce, and innovation

**Cluster 2 (interviewees working in IT)**

- I2: Clinical computer scientist
- I6: Contract and supplier manager
- I9: Clinical computer scientist
- I11: Lead enterprise architect

**Cluster 3 (interviewees who are heads of logistics or supply chain managers)**

- I3: Supply chain manager
- I8: Head of logistics center

#### Confirmatory Stage

The Likert-scale answers of each respondent were first cross-validated with the corresponding top-5 answers to ensure the reliability of the answers. The validation revealed no inconsistency. Subsequently, following Baharmand et al [46], the answers to the Likert-scale questions were averaged at both levels of second-order themes and aggregate dimensions. This resulted in rankings of drivers and barriers at both levels.

### Ethical Considerations

The data processed in this study are not considered to constitute medical research involving human subjects as defined in the 1964 Helsinki Declaration. As such, the statutes of the ethics committees of the authors’ institution indicate that the study, therefore, does not require ethical approval by a review committee. Nevertheless, all the methods described in this study were conducted in accordance with the 1964 Helsinki Declaration and its later amendments or comparable ethical standards. All the interview and questionnaire respondents were informed about the purpose of the data collection and the data processing procedure and consented to participate.

## Results

### Overview

**Figure 1:**
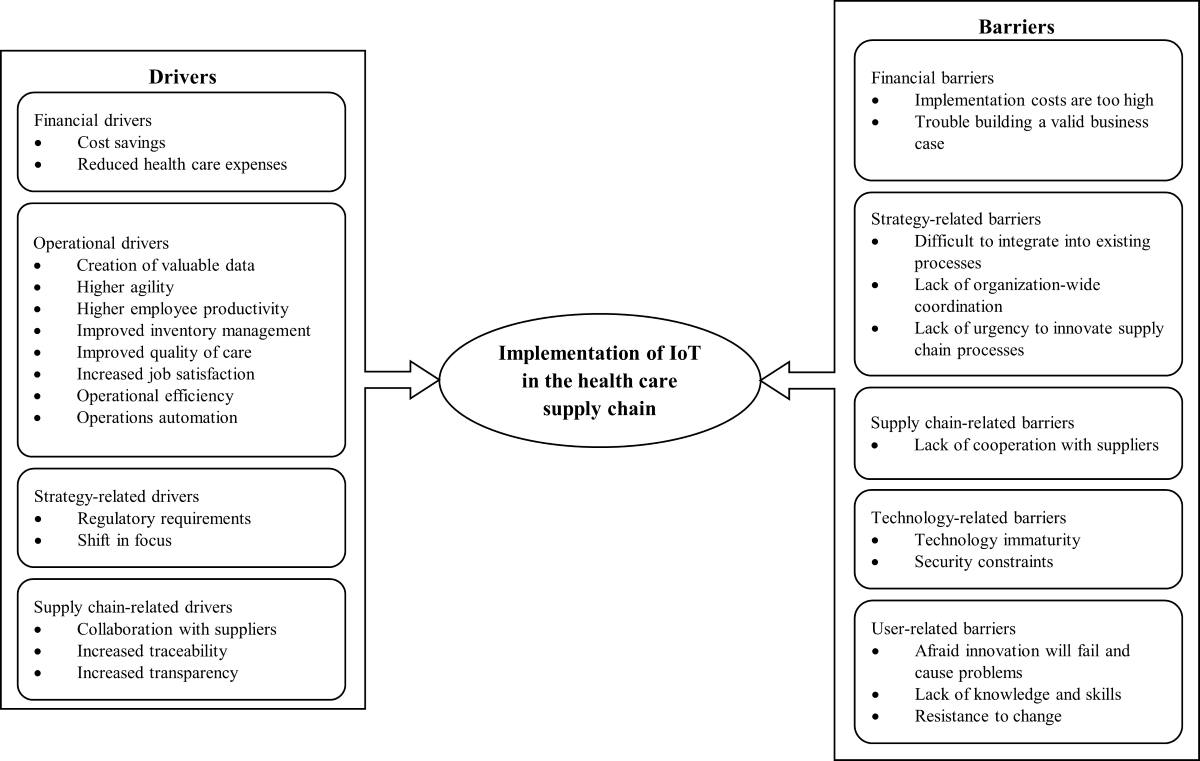
Grounded model of the drivers and barriers to implementing Internet of Things (IoT) in the health care supply chain.

By applying the grounded theory method [50], novel insights were developed on the drivers and barriers to the implementation of IoT in health care supply chains based on the interviews. An overview of the findings can be found in Figure 1. Here, the drivers and barriers have been classified into aggregate dimensions. Both the individual drivers and barriers and the aggregate dimensions will be discussed below. Subsequently, based on the survey results, the importance of each element is determined. The data structures and supporting evidence from the interviews can be found in Multimedia Appendices 6 and 7.

### Exploratory Stage

#### Drivers of Implementing IoT in the Health Care Supply Chain

In this section, the different aggregated driver dimensions are discussed. Table S1 in Multimedia Appendix 8 indicates which interviewees identified which specific drivers.

#### Financial Drivers

Although the implementation of IoT can potentially incur high costs, its use can also lead to major cost savings in various ways. For instance, as fewer employees are needed, hospitals can save personnel costs. Furthermore, better inventory management, enabled by IoT, will lead to fewer rush orders and less expired inventory.

What is really important is that you can save a lot of money in healthcare if you would use track and trace, and that I have tried to articulate as good as possible in the business case.

I6

Eventually, these cost savings can be passed on to patients and taxpayers, resulting in a reduction in health care expenses and insurance costs.

#### Operational Drivers

This aggregate dimension covers a rather large number of drivers. Of these drivers, the creation of valuable data, operational efficiency, improved inventory management, and operations automation were deemed the most important. Using IoT, large amounts of data can be collected, which could potentially provide useful information to improve performance. Also, by implementing IoT, the efficiency of the chain can be increased by automating certain processes, making operations faster and more flexible while reducing human error. IoT can be used to improve inventory management by providing real-time information on inventory levels since many health care facilities currently lack insight into their inventories, leading to unnecessarily high stock levels and out-of-stocks. Furthermore, the opportunity to automate operations using IoT was identified by every interviewee. For instance, the ordering process could be automated, and robots could be used to transport goods within a hospital.

We are working on a pilot with smart warehouse racks. In the room, there are cameras which signal what you take out. They recognize the product based on a QR code and then the smart cameras register the usage and allow for an automatic order.

I3

Other operational drivers reported are improved quality of care, higher employee productivity, increased job satisfaction, and greater agility.

#### Strategy-Related Drivers

First, there are regulatory requirements that encourage management to innovate in order to comply with new regulations or reap the benefits of the loosening of current regulations. From a more internal perspective, organizations also seem to be shifting their focus and strategy more toward innovating within the health care supply chain. Although changes may not always be rapid, the urgency of innovating is increasingly acknowledged, and COVID-19 also played a role in highlighting the importance of supporting processes such as logistics and procurement.

Thanks to COVID-19 it has become apparent that without supplies we cannot provide care. So, it has become less obvious that the supplies are always there.

I3

#### Supply Chain–Related Drivers

The supply chain–related drivers include collaboration with suppliers, increased traceability, and increased transparency. First, the implementation of technologies, such as IoT, may be needed to be able to collaborate effectively with a certain supplier and its information system. IoT can also be used by a hospital to enable further information-sharing throughout the supply chain, with 6 of the 12 interviewees stating that they did not exchange as much information with their suppliers as they would have liked. By sharing information regarding stock levels with suppliers, they can, for example, detect irregularities in demand sooner and respond to them.

Especially with COVID-19, some disruptions you do not detect immediately. There you do miss something. If the supplier would have known that the usage of a certain supply would rise fast, perhaps you could have done something to prevent the shortages.

I7

Furthermore, the need for increased traceability throughout the chain was mentioned by 11 of the 12 interviewees. This would enable the possibility of checking the status of transport to ensure the quality of supplies, and by using track and trace to locate supplies and devices within a health care facility, less time would be spent searching for certain items and fewer goods would be lost. Finally, an increase in transparency was mainly desired in order to get a better insight into the arrival of supplies, not just when supplies are delivered but also what exactly is delivered since orders are often delivered in multiple deliveries without an accompanying order confirmation.

#### Barriers to Implementing IoT in the Health Care Supply Chain

This section elaborates on the aggregate dimensions regarding the barriers to implementing IoT. Table S2 in Multimedia Appendix 8 indicates the interviews in which specific barriers were identified.

#### Financial Barriers

The financial implications of implementing IoT in the health care supply chain were frequently mentioned by interviewees and are therefore considered an important barrier. Hospitals have to work within strict budgets, especially given the significant budget cuts in the health care industry in recent years, which have also affected the supporting processes. Additionally, implementing IoT applications is considered expensive.

You will need to label every good with an RFID tag. For implants that is not a problem, as it already costs a couple of thousand euros, however, in the case of cotton swabs or towels, it becomes too expensive.

I11

The cost challenge is further exacerbated by the fact that there is a rather fragmented budget structure in the health care industry, which makes it difficult to decide who will pay the implementation costs. In addition to the high implementation cost, it is also difficult to build a valid business case since monetizing all the benefits is challenging. For instance, it can be difficult to monetize aspects such as increased job satisfaction, higher quality care, and increased patient safety. Consequently, it is difficult to evaluate if the added value exceeds the costs.

#### Strategy-Related Barriers

Three types of strategy-related barriers were identified: (1) difficulty in integrating into existing processes; (2) lack of internal organizational coordination; and (3) lack of urgency. First, integrating IoT into the existing processes is very challenging due to the lack of standardization within health care processes (eg, differences between general vs specialized hospitals and nonacademic vs academic hospitals) and the high complexity (eg, the large number of suppliers). Second, there is a lack of organization-wide coordination. Hospitals generally consist of many different departments that all work within their own silos.

I think it is a real obstacle that hospitals are really a group of different islands. They are eager to innovate but they are not always capable of organizing, also because they do not have enough knowledge and skills themselves.

I4

In line with this, hospitals typically have a fragmented budget structure and have trouble looking at the larger picture, making it difficult to organize and cross-fund the implementation of IoT. Finally, there is a lack of urgency to innovate supporting processes, such as supply chain management, in general. As a result, the health care supply chain lags considerably behind supply chains in other industries. One possible explanation for this is that supply chain management is not the core business of a hospital; several interviewees perceive that the costs for supply chain management are only a small fraction of the total budget and, therefore, little attention is given to improving these supporting processes.

#### Supply Chain–Related Barriers

The lack of cooperation with suppliers can be seen as both a driver to intensify the collaboration and a barrier since a certain level of cooperation is needed to successfully implement IoT in the supply chain. However, suppliers are often unable or reluctant to cooperate.

The supplier does not always want to tell you that they have problems with their production process because they do not want you to seek an alternative supplier.

I9

#### Technology-Related Barriers

An important barrier that was pointed out by most interviewees is the current technological immaturity found within hospitals. Most health care institutions are lagging behind what is seen elsewhere when it comes to data collection and analysis, and their infrastructure is often not ready to facilitate technologies such as IoT. A total of 2 interviewees also indicated that several other technological innovations have higher priority than IoT because they are less costly and easier to implement. Another technology-related barrier is security constraints. As the health care industry is a highly regulated market, any violation of these regulations (eg, data breaches) can place great liability on hospitals.

We have to obey those kinds of rules. The hospital is of course really afraid that if something goes wrong regarding privacy and the General Data Protection Regulation, we get high fines. And if something goes wrong with security and our systems go down, it will be printed in the papers that care delivery was jeopardized.

I6

Although the implementation of IoT for supply chain purposes would generally not involve patient information, there are other security implications besides privacy concerns that discourage the use of IoT. For instance, a disturbance in the IT network could ultimately have implications for patients’ safety.

#### User-Related Barriers

These barriers relate to the users of IoT, such as the directors and managers that must approve the implementation of IoT, the logistics personnel, and the health care personnel. First, some users inherently fear that the innovation will fail and generally cause problems due to their lack of trust in its success. Such fears can be caused by the failure of previous innovations. Furthermore, there is also an apparent lack of knowledge and skills needed to implement technological innovations such as IoT, with many potential users unaware of the possibilities of IoT in the supply chain. However, even if these opportunities could be identified, hospitals often lack the ability to implement them. This may relate to employees’ resistance to change.

The consequences of the implementation of IT on your work processes and the consequences of that on the professional identity of employees is something that we really struggle with. So, we see a lot of resistance to the introduction of these kinds of things.

I11

Besides professional identity, employees tend to be satisfied with the way things are and prefer to stay in that comfortable environment. Finally, resistance also comes from the fear of being replaced by technology. For instance, if logistical tasks are automated, fewer employees will be needed to perform those tasks.

#### Cluster Analysis

The cluster analysis of the interview transcriptions revealed that there were drivers for implementing IoT technology that were consistently found across all clusters, including improved inventory management and operations automation (Table S1 in Multimedia Appendix 8). It is noteworthy that the first cluster, in which the interviewees were mainly executing projects, mentioned improving care quality more frequently as a driver, whereas this driver was not stressed as much by the other clusters. Furthermore, similarities and differences were observed in the barriers identified by each cluster (Table S2 in Multimedia Appendix 8). Across all clusters, a lack of cooperation with suppliers was only mentioned to a limited extent as a barrier. Also, it was mainly IT professionals who referred to the difficulty of building a valid business case and the security constraints as important barriers. Furthermore, the cluster of interviewees who are primarily executing projects identified the importance of the lack of organization-wide coordination more frequently than interviewees in the other clusters.

### Confirmatory Stage: Ranking of Drivers and Barriers

**Figure 2:**
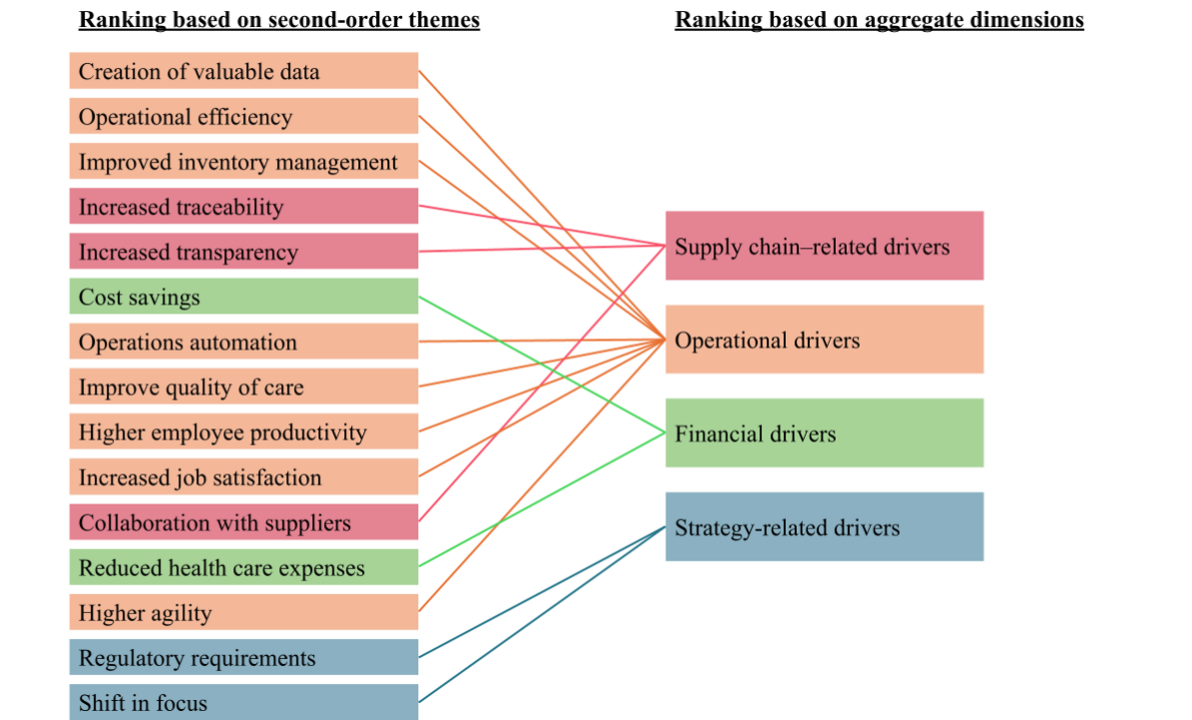
Rankings of drivers.

**Figure 3:**
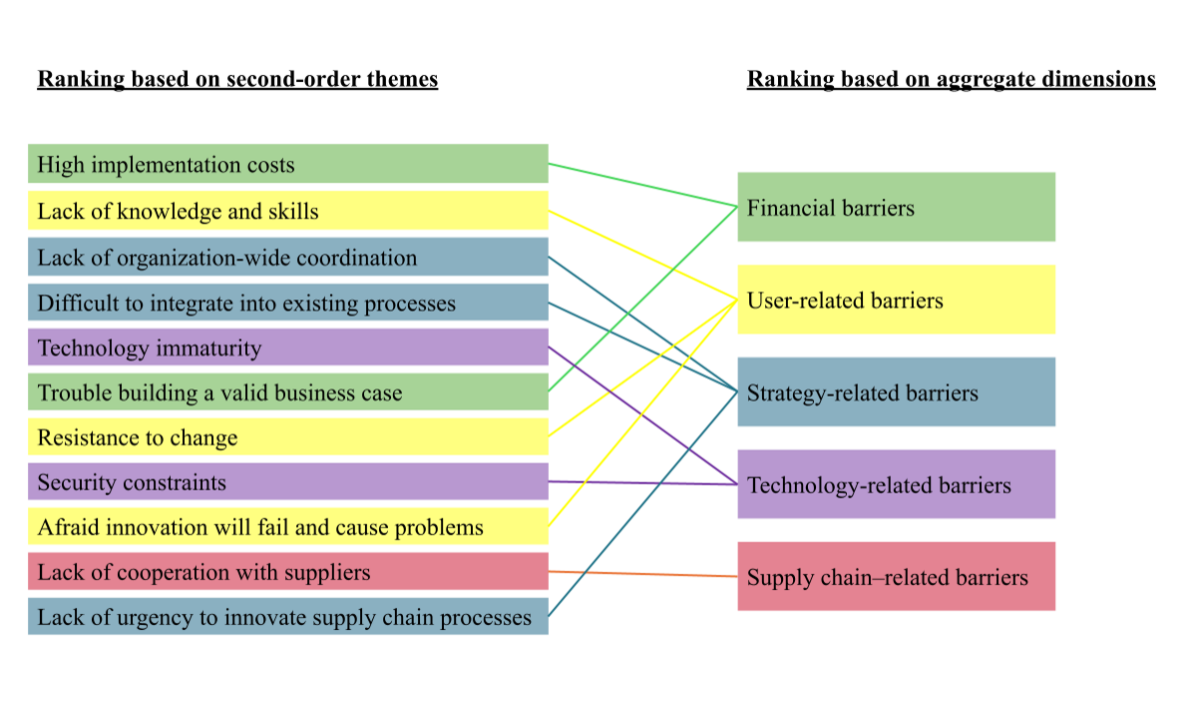
Rankings of barriers.

Based on the survey results, the individual second-order drivers and barriers identified from the earlier interviews have been ranked in importance (Table 2). From the average scores of the second-order drivers within an aggregate dimension, it can be seen that the supply chain–related drivers are deemed the most important, followed by the operational drivers, the financial drivers, and the strategy-related drivers (see Figure 2). Similarly, regarding the barriers, the financial barriers were identified as the most important, followed by user-related barriers, strategy-related barriers, technology-related barriers, and supply chain–related barriers (see Figure 3). This ranking is generally in line with the frequency with which they appear in the interviews (Tables S1 and S2 in Multimedia Appendix 8). One notable exception was that while the lack of urgency was mentioned in many interviews, it was ranked relatively lowly, suggesting that this barrier is prevalent but not strong.

**Table 2:** Drivers and barriers ranked by order of importance.

Rank	Drivers	Rank	Barriers
1	Creation of valuable data	1	Implementation costs are too high
2	Operational efficiency	2	Lack of knowledge and skills
3	Improved inventory management	3	Lack of organization-wide coordination
3	Increased traceability	4	Difficult to integrate into existing processes
5	Increased transparency	4	Technology immaturity
6	Cost savings	6	Trouble building a valid business case
7	Operations automation	6	Resistance to change
8	Improved quality of care	8	Security constraints
9	Higher employee productivity	9	Afraid innovation will fail and cause problems
10	Increased job satisfaction	10	Lack of cooperation with suppliers
11	Collaboration with suppliers	11	Lack of urgency to innovate supply chain processes
11	Reduce health care expenses	N/A^a^	N/A
13	Higher agility	N/A	N/A
14	Regulatory requirements	N/A	N/A
15	Shift in focus	N/A	N/A

^a^Not applicable.

## Discussion

### Principal Findings

This study has empirically investigated the drivers and barriers to implementing IoT in health care supply chains and, based on a survey, proposed a ranking of the perceived drivers and barriers in terms of their importance. Based on the interview and survey data, we found that financial, operational, strategy-related, and supply chain-related drivers are key to implementing IoT. Meanwhile, financial, strategy-related, supply chain–related, technology-related, and user-related barriers restrain hospitals from implementing IoT more widely in their supply chains. By ranking the drivers and barriers, we provide an overview of their relative significance and the general situation of the health care industry regarding IoT adoption. This ranking can provide guidance on which drivers and barriers to first address when seeking to implement IoT technologies in health care.

### Comparison With Previous Work

Key drivers of IoT in other sectors have been shown to be improving operational processes and inventory management, realizing environmental and social sustainability, risk management, cost reduction, and supply chain transparency [10,27,48]. Widely identified barriers to implementing IoT in supply chain management are technical set-up costs, training costs, resistance to change, lack of coordination, security concerns, and ongoing support costs [10,27,48]. Technical challenges include limited interoperability between devices and the management of data collection, storage, analysis, and exchange. There have been some studies in a health care setting that have highlighted facilitating and limiting conditions for applying IoT technologies, as well as other technologies, that could contribute to supply chain transparency and equipment traceability. Focusing on clinical applications, Kelly et al [28] highlight the importance of accessible and secure technology, supported by appropriate policies and regulations. Zhu et al [51] show a trend toward greater application of tracing technologies in supply chain management, although the drivers and barriers to such uses have not been highlighted. As such, this study is one of the first to empirically investigate the drivers and barriers to implementing IoT in health care supply chain management. Moreover, we are the first to rank the importance of these drivers and barriers as perceived by potential IoT technology users and related stakeholders. In the following sections, we reflect on the drivers and barriers that appear key given the current challenges and recent developments in health care.

#### IoT for Resilient Health Care Supply Chains

Given the disastrous consequences of the COVID-19 pandemic on health care equipment and medication availability in many countries, improving resilience has become a key goal in health care development. Hence, it is not surprising that several of the highly ranked drivers are linked to increasing supply chain resilience. The highest-ranked driver in this study is data availability. As IoT enables the collection and processing of large quantities of real-time data [11], it creates the possibility for fast and efficient information sharing [12-14], which can create a more flexible supply chain [15]. This was reflected in the operational and supply chain–related drivers. Other research shows the importance of IoT in establishing better links with subtier vendors, thereby gathering real-time progress and inspection data from them and also enabling continuous monitoring of quality during production [10]. Examples from a global supply chain perspective are a more accurate insight into each warehousing and transportation step, supporting incident-related queries of equipment or pharmaceutical production, and preventing or identifying potential theft and forgeries of pharmaceuticals. Hence, it is not surprising that supply chain transparency was highly ranked as a driver of IoT, and indeed, it is generally assumed to increase visibility throughout the supply chain, thereby signaling internal and external irregularities at an early stage and providing warnings to organizations [10,16,17,19]. In this way, IoT can enable a quicker response and mitigating actions in response to disruptions, thereby improving the speed and efficiency of the entire global supply chain [16,17].

#### Care Provider Governance

This study found that while cost savings may drive IoT implementation, high implementation costs and the difficulty of generating a valid business case were frequently mentioned barriers. Making cost savings visible is not an uncommon problem and can be particularly challenging in the health care industry [52-55]. In part, this is because hospitals have many different cost centers and budgets, and investments often have to fall within a single department [53-55]. Meanwhile, the benefits of some investments, such as the implementation of IoT, may extend well beyond the funding department alone, and, as such, it can be difficult to find a department willing to make the investment. This issue is also an aspect of the identified strategy-related barrier of “lack of organization-wide coordination.” Thus, moving beyond “silo thinking,” and adopting a more organization-wide perspective on IoT implementation would aid in identifying all the benefits that could be gained, which would then make it easier to build a valid business case and overcome organizational resistance.

#### Knowledge and Awareness to Facilitate Change Management

Even though the urgency to innovate in health care is increasingly recognized, resistance to change remained very evident in this study. Others have acknowledged that values related to risk-taking and entrepreneurial behavior remain scarce [54]. Part of the resistance to change may come from the fact that professional identity is of great importance to health care workers [56]. This identity may be affected if tasks that are currently a large part of their work are reallocated to other workers or even fully automated through the use of IoT. Therefore, to successfully implement change, employees should be involved by being given the opportunity to express their wishes and fears [57]. In addition, a more gradual strategy could be adopted where implementation starts with small pilots and is slowly scaled up until a system-wide rollout is achieved [58]. Hayes [59] proposed creating a sense of urgency by presenting a vision of a more desirable future and informing employees with state-of-the-art knowledge. This may also overcome our observation that several employees consider supply chain management-related costs relatively low, in contrast to what is commonly reported [25,26]. In short, improving knowledge among employees can weaken the opposition and motivate them to let go of the status quo.

### Managerial and Policy Implications

Several lessons can be drawn from this study. First, to overcome the described challenging barriers to IoT implementation, health care managers and policy makers need to recognize the significance of supply chain management in the health care industry, especially considering the challenges faced during the COVID-19 pandemic. The findings highlight the potential of IoT to address supply chain disruptions by enabling a more resilient and efficient supply chain. Therefore, managers should prioritize investing in IoT technologies and solutions to enhance supply chain operations.

The identification and ranking of drivers and barriers provide valuable insights for policy makers. Although regulators such as the European Union pay much attention to increasing the traceability of medical equipment [60,61], our research has identified several challenges. Policy makers should be aware of the high implementation costs of IoT technologies, which are ranked as the most important barrier. Current financial incentives in most health care systems, however, do not reward suppliers and care providers for realizing better care outcomes while reducing costs. Hence, policy makers should steer toward value-based health care funding [62], which aligns incentives between the various health care supply chain entities and drives innovative solutions such as IoT.

The mentioned updated European Union medical equipment regulation provides a cause for strengthening communication between policy makers, suppliers, and health care providers. Care provider managers should explore cost-effective IoT solutions and develop strategies to justify the investment in IoT by demonstrating its long-term benefits, such as cost savings and improved efficiency. Furthermore, health care provider leaders should consider the operational and technological aspects of IoT implementation. They should ensure that the organization has the necessary infrastructure, knowledge, and willingness to support IoT integration into the supply chain. Training programs developed and continuous education provided by joint stakeholders can help health care professionals and support staff adapt to the changes brought by the IoT and use the technology effectively [60].

### Strengths, Limitations, and Future Research Directions

This study contributes to the literature on the implementation of IoT in health care supply chains as it is among the first to provide empirical evidence on the drivers and barriers of IoT related to supply chain management in health care. Hereby, we extend the insights provided by the conceptual work of Kelly et al [28]. This is also the first paper to rank the drivers and barriers. We have done so by collecting insights from professionals considered knowledgeable on supply chain management in this section and taking a broad scope when obtaining their input. Using a mixed-methods approach, the study has generated key categories of drivers and barriers, providing structure and tools for managers and policy makers.

We also recognize some limitations of this study, creating opportunities for future research. First, the scope of this study is limited, as it only includes health care organizations in the Netherlands. The external validity of the study could be increased by including a larger number of cases, including those from other countries. Furthermore, the study was set up to understand a care provider’s perspective, so the inputs were mainly from hospital staff. Innovations such as IoT are likely to also be driven by medical equipment or pharmaceutical suppliers, and, hence, one could investigate what they consider to be the drivers of and barriers to IoT.

The presented ranking of drivers and barriers was based on the interviews conducted and responses to a small-scale survey. Although the final ranking presented is highly consistent with both data sources, this is the first attempt to map the perceived importance of these items. A larger survey combined with a Delphi panel could further validate the ranking.

Furthermore, in this preliminary study, no distinction was made between implementation phases. Damanpour and Schneider [63] distinguished three phases in the adoption of innovation: (1) initiation, (2) adoption decision, and (3) implementation of the innovation. In following up our research, these phases could be addressed separately, thereby providing a deeper understanding of the drivers and barriers in each implementation phase. Following this, guidelines could be developed as to how the drivers can best be exploited and how the barriers could be lowered.

### Conclusions

Given the current scarcity of personnel and financial resources in many health care sectors, innovative technological solutions are pivotal in ensuring the delivery of accessible and high-quality care. Particularly when it comes to the large costs incurred in the supply chain management of various resources, including equipment and pharmaceuticals, solutions such as IoT have the potential to contribute to creating the necessary budgetary space. The barriers identified in this study explain why the health care sector lags behind other sectors where IoT technology is already more prominent. At the same time, the study reveals that many employees of health care providers recognize the benefits of IoT applications in improving health care supply chain management, thereby showing that this sector has the potential to catch up in this development. The ranking of drivers and barriers to IoT implementation provides guidance for practitioners as to which are the most urgent to address. Also, it highlights what is perceived as the most important drivers, which health care leaders could emphasize in pushing IoT development forward.

## Appendix

Multimedia Appendix 1 Overview of relevant literature.

Multimedia Appendix 2 Interview guide.

Multimedia Appendix 3 COREQ checklist.

Multimedia Appendix 4 Survey design.

Multimedia Appendix 5 Descriptive statistics of survey respondents.

Multimedia Appendix 6 Data structure drivers to implementing Internet of Things in the health care supply chain.

Multimedia Appendix 7 Data structure barriers to implementing Internet of Things in the health care supply chain.

Multimedia Appendix 8 Tables S1 and S2.

## Abbreviations

COREQ: Consolidated Criteria for Reporting Qualitative Research
IoT: Internet of Things
TOE: Technology-Organization-Environment

## Data Availability

The data sets generated during and analyzed during this study are available from the corresponding author upon reasonable request.
