# Supplementary material for: Drivers and Barriers to Implementing the Internet of Things in the Health Care Supply Chain: Mixed Methods Multicase Study
Source: J Med Internet Res. 2023 Sep 20;25:e48730. doi: 10.2196/48730 (PMC10551782; doi:10.2196/48730)
Supplement: Multimedia Appendix 1 [file jmir_v25i1e48730_app1.docx]

**Multimedia Appendix 1.** Overview of relevant literature

|  | **Topic of interest** | **Use cases** | **Drivers** | **Barriers** |
| --- | --- | --- | --- | --- |
| *IoT in supply chain management* | |  |  |  |
| Aryal et al. (2020) | The emergence of IoT in SCM | RFID for track and trace, robotics and dynamic pricing | Improvement of operational processes, realizing environmental and social sustainability, inventory management optimization and risk identification, mitigation, response and recovery | Data security and privacy challenges, need to develop new supply chain strategies and models |
| Sodhi et al. (2022) | User's experiences with emerging supply chain technologies including IoT |  | Operational efficiency, performance of existing system, employee productivity, real-time capability, transparency and cost-effectiveness as most important goals and affordances | Technical setup cost, training cost, resistance to change, lack of organization-wide coordination, security concerns and ongoing support cost as most important constraints |
| Ben-Daya, Hassini and Bahroun (2019) | Aims to explore the impact of IoT on supply chain management | Link with sub-tier vendors, collection of supply chain data, inventory tracking, information-sharing, autonomous decision-making, i.a. | Increased (real-time) visibility throughout chain, reduced lead times, reduce costs, increase customer satisfaction, increased inventory accuracy, i.a. | Security and privacy issues and interoperability |
| Birkel and Hartmann (2019) | Challenges and risks of IoT in supply chain management |  |  | High costs, privacy concerns, unknown profitability, security issues, hard- and software limitations, trust issues, need for high collaboration and data exchange, lack of knowledge, concerns of employees, i.a. |
| *IoT in healthcare industry* | |  |  |  |
| Chanchaichujit et al. (2019) | Discusses benefits of and provides a framework for implementing Industry 4.0 technologies, including IoT, in healthcare services | Clinical care and remote control | Governmental initiative, patients demanding better services, emergence of integrated care and the current data revolution | Interoperability, data management, privacy and security |
| Tortorella et al. (2020) | Investigates trends, challenges and theoretical gaps regarding the implementation of Healhcare 4.0, including the use of IoT | Electronic health record systems, mobile health applications, health treatment, personnel management and scheduling systems | Improved diagnosis and care services, improved transparency, lower lead times, foster collaboration, cost reduction and improved support to training and education | Poor ICT-infrastructure, Information security risks, patents, lack of skills, costs of implementation, misalignment with strategy, systems heterogeneity and regulatory changes |
| Dantu, Dissanayake and Nerur (2021) | Identify main themes in academic literature regarding IoT applications in healthcare | Remote vital signs monitoring, medication management and healthcare solutions via smartphone | Time savings, enables proactive intervention and facilitate real-time, high-quality care | Challenges regarding privacy and security |
| Rakshit, Nath and Pal (2020) | The application of IoT in healthcare | Remote patient monitoring, asset monitoring, improved drug usage, healthcare charting and improving emergency care by sending information up front | Simultaneous reporting and monitoring, end-to-end connectivity, data analysis, tracking, on-time warning system and remote medical assistance | Data safety, integration of devices and protocols, data overload and costs |
