# Supplementary material for: Drivers and Barriers to Implementing the Internet of Things in the Health Care Supply Chain: Mixed Methods Multicase Study
Source: J Med Internet Res. 2023 Sep 20;25:e48730. doi: 10.2196/48730 (PMC10551782; doi:10.2196/48730)
Supplement: Multimedia Appendix 2 [file jmir_v25i1e48730_app2.docx]

**Multimedia Appendix 2.** Interview guide

| **Interview guide** | | |
| --- | --- | --- |
| Organization: | |  |
| Interviewee: | |  |
| Interviewer: | |  |
| Date: | |  |
| Duration of interview: | |  |
| **Introduction**   - Thank participant for cooperating - Ask for approval to record the interview - Explain the research   - This study aims to identify the drivers and barriers to implementing Internet of Things in the healthcare supply chain. Questions will be asked regarding:     - The organization and your role within the organization     - The use of Internet of Things (IoT) within the organization     - Drivers and barriers of the use of IoT - The interview will take approximately 45 minutes. - Ask whether there are questions before continuing | | |
| **Questions** | | |
| 1. | *Interviewee and organization (X):*   - What is your role within X? - What is your connection to supply chain management? - How large is X in terms of employees and patients? - How many different suppliers does X have? - To what extent do you share information or are you in contact with your suppliers?   - E.g. information regarding inventory, circumstances of storing and transporting supplies, delivery agreements, etc. - What is X’s overall objective in handling supply chain disruptions? - How would you describe the attitude of X towards digitization and technological innovation? - What do you know about Internet of Things yourself? | |
| 2. | *Use of IoT*:   - Does X currently make use of IoT organization-wide? Can you elaborate? - Does X currently make use of IoT for supply chain management specifically? Can you elaborate? | |
| 3. | *If IoT is being used within the supply chain:*   - *Drivers*   - What were the main drivers for implementing IoT in the supply chain?     - E.g. costs, market expectations, pressure from suppliers, efficiency, real-time monitoring of supplies, getting insight, etc.   - What stakeholders are involved in this process?     - E.g. external suppliers, departments within the hospital, other hospitals, etc.   - Who initiated the project of implementing IoT?     - E.g. which department or even specific function   - What was the perception of other employees about the implementation of IoT? - *Barriers*   - What were the barriers you encountered when implementing IoT?   - How did you overcome these barriers?     - E.g. did you receive help from other stakeholders) - *Other*   - Would you say the implementation of IoT is successful?     - Yes: What were the benefits that made you perceive the use of IoT as successful?     - No: What are the reasons why you do not perceive the implementation of IoT as successful? Have you perceived any benefits at all?   - How was the relationship with other stakeholders in the supply chain influenced as a result of the implementation of IoT?     - E.g. regarding trust, the extent of information-sharing, etc.   - Do you think there are consequences for the quality of patient care caused by the implementation of IoT?   - Are there things you would have done differently when looking back at the implementation phase? | |
| 4. | *If IoT is not being used within the supply chain:*   - Have there been initiatives to implement IoT in the past?   - If yes: Can you tell why these initiatives have not been implemented permanently?   - If no: What do you think is the reason that there are no such initiatives at X yet?     - E.g. costs, resistance, security issues, lack of knowledge, etc. - How could you overcome these barriers? - In your opinion, for what kind of applications could IoT be utilized in your organization? | |
| **Conclusion of interview** | | |
| A. | Is there anything you would like to add that did not come up yet during the interview? | |
| B. | Do you have any questions? | |
| C. | Ask for the possibility of a follow-up meeting in case of any unclarities | |
| D. | Ask whether the interviewee knows other potential interviewees leading from this interview | |
| E. | Ask whether the interviewee is willing to provide feedback on a summary of this interview | |
| F. | Thank participant again for cooperation | |
