# Supplementary material for: Drivers and Barriers to Implementing the Internet of Things in the Health Care Supply Chain: Mixed Methods Multicase Study
Source: J Med Internet Res. 2023 Sep 20;25:e48730. doi: 10.2196/48730 (PMC10551782; doi:10.2196/48730)
Supplement: Multimedia Appendix 4 [file jmir_v25i1e48730_app4.docx]

**Multimedia Appendix 4.** Survey design

| **Drivers and barriers of IoT in the healthcare supply chain** | |
| --- | --- |
| ***Introduction***    Dear participant,    Thank you for participating in this survey! Below you will find a brief instruction. The survey will take approximately 10 minutes to complete.    **Background**. Through this survey, facilitating and impeding elements for the use of the Internet of Things (IoT) in the medical supply chain are being explored. An initial exploratory survey has already identified several elements based on interviews. This widely distributed survey aims to validate the findings, and to determine which elements are perceived as most important.    **Use of data**. No personally identifiable data will be requested. All data will be kept confidential and will be published only in aggregate form. Thus, no individual responses will be listed. The insights gained from this survey will be shared with participants. By participating in the survey, you agree that the completed data may be published in an academic journal.  **Internet of Things (IoT)**. IoT can be defined as the network of devices that interact, connect and exchange data with other devices or systems via Internet connections without human intervention. Examples of IoT in everyday life are smart devices such as a smart watch or a smart thermostat that you can control remotely. An example of IoT in the supply chain is that up-to-date information regarding inventory levels can be continuously shared with suppliers allowing earlier response to shortages or the ability to constantly monitor product quality during the production process. | |
| Questions | |
| 1 | What is your age?   - Younger than 25 years of age (1) - 26-35 years (2) - 36-45 years (3) - 46-55 years (4) - 56-65 years (5) - Older than 65 years of age (6) |
| 2 | Within what type of organization - which is part of the medical supply chain - do you work?   - Healthcare facility (1) - Logistics service provider (2) - Supplier of medical devices or medicines (3) - Other, namely: … (4) |
| 3 | What is your role within your organization?   - Caregiver (1) - Logistics employee (2) - Employee purchasing department (3) - Employee innovation department (4) - Employee sales department (5) - Central management or central support function (6) - Other, namely: … (7) |
| 4 | How many years have you been working in your current position?   - Less than 1 year (1) - More than 1 year, but less than 5 years (2) - More than 5 years, but less than 10 years (3) - More than 10 years, but less than 15 years (4) - More than 15 years, but less than 20 years (5) - More than 20 years (6) |
| 5 | To what extent do you hold a managerial position?   - None (1) - Leadership at the team level (2) - Department level leadership (3) - Management-level leadership (4) |
| 6 | *Display this question if Q5 != None*  How many people work under your supervision?   - 1-9 (1) - 10-24 (2) - 25-49 (3) - 50-99 (4) - 100 or more (5) |
| 7 | How do you experience the workload?   - Very low (1) - Low (2) - Somewhat low (3) - Not low, not high (4) - Somewhat high (5) - High (6) - Very high (7) |
| 8 | To what extent are you familiar with the term Internet of Things (IoT)?   - Not familiar with (1) - Heard of, but no knowledge about (2) - Somewhat familiar with (3) - Well familiar with (4) - Very familiar with (5) |
| 9 | Are IoT applications used, or planned to be used, within your organization?   - No applications or plans (1) - Limited applications and/or plans (1 or 2) (2) - Several applications and/or plans (up to 5) (3) - Many applications and/or plans (5 to 10) (4) - Very many applications and/or plans (10 or more) (5) |
| 10 | Can you name one or more applications of IoT within your organization?   - No (1) - Yes, namely: … (2) |
| 11 | Select the 5 drivers that you believe are most relevant to the use of Internet of Things in the healthcare supply chain.   - Collaboration with suppliers - Cost savings - Creation of valuable data - Higher agility - Higher employee productivity - Improved inventory management - Improved quality of care - Increased job satisfaction - Increased traceability - Increased transparency - Operational efficiency - Operations automation - Reduce healthcare expenses - Regulatory requirements - Shift in focus |
| 12 | From your perspective, please answer per item the statement, “the listed item is a driver for the use of Internet of Things in the medical supply chain”. Choose from the following options.   - Completely disagree (1) - Disagree (2) - Somewhat disagree (3) - Disagree or disagree (4) - Somewhat agree (5) - Agree (6) - Completely agree (7) |
| 13 | Are there any other motivations not identified in the question above? |
| 14 | Select the 5 barriers that you believe are most relevant to the use of Internet of Things in the healthcare supply chain.   - Afraid innovation will fail and cause problem - Difficult to integrate into existing processes - Implementation costs are too high - Lack of cooperation with suppliers - Lack of knowledge and skills - Lack of organization-wide coordination - Lack of urgency to innovate supply chain processes - Resistance to change - Security constraints - Technology immaturity - Trouble building a valid business case |
| 15 | From your perspective, please answer per item the statement, “the listed item is a barrier to the use of Internet of Things in the medical supply chain”. Choose from the following options.   - Completely disagree (1) - Disagree (2) - Somewhat disagree (3) - Disagree or disagree (4) - Somewhat agree (5) - Agree (6) - Completely agree (7) |
| 16 | Are there any other barriers not identified in the question above? |
