# Supplementary material for: Drivers and Barriers to Implementing the Internet of Things in the Health Care Supply Chain: Mixed Methods Multicase Study
Source: J Med Internet Res. 2023 Sep 20;25:e48730. doi: 10.2196/48730 (PMC10551782; doi:10.2196/48730)
Supplement: Multimedia Appendix 5 [file jmir_v25i1e48730_app5.docx]

**Multimedia Appendix 5.** Descriptive statistics of survey respondents

| **Variable** | **Frequency** |
| --- | --- |
| *Age in years* |  |
| < 25 | 1 |
| 26 – 35 | 2 |
| 36 – 45 | 5 |
| 46 – 55 | 8 |
| 56 – 65 | 10 |
| > 65 | 0 |
| *Employer* |  |
| Healthcare organization | 7 |
| Logistics provider | 3 |
| Supplier of medical supplies | 11 |
| Other | 5 |
| *Years of experience in current position* |  |
| Less than 1 year | 4 |
| More than 1 year but under 5 years | 7 |
| More than 5 years but under 10 years | 4 |
| More than 10 years but under 15 years | 3 |
| More than 15 years but under 20 years | 4 |
| More than 20 years | 4 |
| *Level of leadership* |  |
| None | 7 |
| Leading at team level | 5 |
| Leading at department level | 7 |
| Leading at board level | 7 |
| *Familiarity with Internet of Things* |  |
| Very unfamiliar | 0 |
| Unfamiliar | 4 |
| Somewhat familiar | 7 |
| Familiar | 13 |
| Very familiar | 2 |
| *Application of IoT within respondent’s organization* |  |
| No applications | 2 |
| Limited applications or plans to implement (1 or 2) | 11 |
| Various applications or plans to implement (3 – 5) | 8 |
| Many applications or plans to implement (5 – 10) | 5 |
| Numerous applications or plans to implement (more than 10) | 0 |
