# Supplementary material for: Drivers and Barriers to Implementing the Internet of Things in the Health Care Supply Chain: Mixed Methods Multicase Study
Source: J Med Internet Res. 2023 Sep 20;25:e48730. doi: 10.2196/48730 (PMC10551782; doi:10.2196/48730)
Supplement: Multimedia Appendix 6 [file jmir_v25i1e48730_app6.docx]

**Multimedia Appendix 6.** Data structure drivers to implementing IoT in the healthcare supply chain

| **First-order concept** | **Second-order theme** | **Aggregated dimension** |
| --- | --- | --- |
|  | **Cost savings** | Financial drivers |
| Cost savings | **I1**: The most important driver for the hospital is cost reduction. |  |
|  | **I6**: What is really important is that you can save a lot of money in healthcare if you would use track and trace, and thát I have tried to articulate as good as possible in the business case. |  |
|  | **I10**: If malfunctioning of the freezer is signaled in an early stage, it is prevented that all food has to be thrown away. |  |
|  | **I11**: We can realize an enormous cost saving by replacing all the surgical assistants from wage scale 9 by logistics employees in scale 3 or 4. |  |
|  | **Reduced healthcare expenses** |  |
| Reduce healthcare expenses for patients | **I4**: It is in the interest of society that medical expenses are kept to a minimum. Every month citizens pay a vast amount of money for their health insurance and this money should be spent responsibly. |  |
|  | **I9**: If you look at the innovations that have been done in the healthcare industry for the past 40 years. This way it will become unaffordable to continue to provide care. Computers are cheap so we have to digitalize. |  |
|  | **Creation of valuable data** |  |
| Creation of useful data | **I10**: In my opinion, it generates data that can help to improve your service. | Operational drivers  Operational drivers (continued)  Operational drivers (continued)  Operational drivers (continued)  Operational drivers (continued)  Operational drivers (continued) |
|  | **I10**: It can be noticed that increased interest is shown from other departments for what we do, which is the generation of data. |  |
| Gather insight in supply chain | **I3**: The main driver for the supplier is to gather insight into the supply chain. |  |
|  | **I7**: We are clearly putting forward that we want to move in this direction because we want more insight in order to be able to justify ourselves in certain matters. |  |
| Insight in actual usage | **I4**: Having insight into the actual usage is something that the hospitals that I have spoken with up until now really desire. |  |
|  | **I7**: In the end, you want to help the customer in the best way possible. However, you miss something really important. We do not know when something is out of stock and when we have to help and refill. |  |
|  | **Higher agility** |  |
| Ability to signal irregularities in supply chain and act adequately | **I3**: By getting more insight in the supply chain, the supplier can increase its grip on demand and is able to better respond to sudden increases in demand. |  |
|  | **I9**: If you have certain identifiers throughout the supply chain certain alerts can be triggered if irregularities are signalled. As a result, you can approach the situation more calmly and prevent panic situations. |  |
|  | **I10**: Through optimization of your process you can keep goods available, and you are able to react quicker to irregularities. |  |
|  | **I11**: Through optimization of your process you can keep goods available and are able to react quicker to irregularities. |  |
| Increased flexibility | **I4**: You can become more flexible in providing care and the deployment of personnel and consequently lower lead times for patients. |  |
|  | **I11**: It is easier to relocate personnel if processes are standardized and automated. |  |
|  | **Higher employee productivity** |  |
| Better deployment of personnel | **I2**: It is the case that many surgical assistants have to gather the set of instruments needed for surgery from storage themselves. However, they are expensive employees and they are becoming increasingly difficult to find. So, you actually want logistic workers to perform those tasks for them. Thus, by automating some of the work and outsourcing some tasks to logistic workers, surgical assistants can keep themselves occupied with the surgery. |  |
|  | **I5**: We want to deploy our personnel better, thus for the tasks they were educated for. So, logistic workers perform logistic tasks and healthcare workers perform healthcare work. |  |
|  | **I9**: We have been saying it for a while but we are going to have to deliver the same amount of high-quality care with fewer healthcare workers so the labor productivity must increase. |  |
|  | **I10**: We have a tunnel of 250m below the hospital, which results in quite some travel time for employees when they are bringing supplies to another part of the hospital. This could also be performed by robots and as such the employee can save time. |  |
|  | **I12**: We want to give back time to the healthcare personnel so they can spend more time in guiding new patients or comforting patients and their families. |  |
| Less personnel needed | **I1**: Currently, someone has to go to all inventory rooms with a scanner two or three times a week but by automating parts of inventory management this is not necessary anymore. Thus you need fewer people. |  |
|  | **I5**: We have a large number of employees who are all performing manual tasks which could be performed much easier digitally. |  |
|  | **I7**: Even if we could manage to take away the people in the process of picking up the garbage and that kind of thing. That would already make a large difference. |  |
| Tackle labor shortage problems | **I4**: Besides, we experience enormous labor shortages so we want to decrease the work pressure on the healthcare professionals. |  |
|  | **I8**: On the other hand you see that it is becoming increasingly difficult to find employees whilst you also have to carry out work at night for which you do not want to employ people. |  |
|  | **I12**: There is a large labor market shortage, which demands healthcare institutions to transform. |  |
|  | **Improved inventory management** |  |
| Ability to better predict actual use | **I1**: You can now estimate the actual usage better because you know when you are going to be out of stock. Often it is the case that healthcare workers discover we are out. Then a rush order is placed and that disrupts your process. |  |
|  | **I4**: Because there is barely any insight into the actual usage and inventory management, hospitals often have to place rush orders. |  |
| Better availability of supplies | **I1**: It has advantages for both us and the hospital. Fewer disruptions and better availability of supplies. |  |
|  | **I2**: I am not sure how small they are and how easy it is to tag things and link them to share data but for heart valves and implants it is really useful to have the inventory in order because you will need certain things for the operation. |  |
|  | **I6**: I recently had a talk with a department manager and he indicated that inventory management and the department were not aligned well. What then happens is that if the surgeon wants to perform a planned surgery and the necessary goods are not available, then the patient needs to be sent home. |  |
| Decrease inventory levels and expired inventory | **I5**: We want optimal inventory levels. Currently, we have enormous inventory levels whilst that is not necessary at all. |  |
|  | **I7**: It is also the case that we have large inventories but our suppliers have an even larger inventory. And perhaps there is a supplier before that which has an even larger inventory. We have received goods which approached their expiry date whilst they normally have a shelf life of three or four years. Then I am asking myself how you can possibly achieve that. |  |
|  | **I11**: We will start to think about the construction of a new OR and reconstruction of our current OR and there we want to allocate as much space as possible to the primary process and thus as little space as possible to inventory and that kind of things. |  |
|  | **Improved quality of care** |  |
| Decrease lead times | **I4**: I think you can decrease the throughput time a lot and as a result, the patient has less time to get sicker if you are treating someone with cancer for instance. |  |
|  | **I6**: If it is insightful whether the necessary goods and equipment are available with track and trace, it will save patients waiting time and you need less equipment which saves investments. |  |
| Improvement of quality of care | **I3**: If the pilot is ever going to succeed and we get smart warehouse racks like the refrigerator from Samsung which knows exactly what is in it and automatically orders cheese and milk if they are empty, that would save me a lot in quality and efficiency. |  |
|  | **I4**: Ultimately, hospitals want to deliver the best care possible to patients because patients have to choose for them. |  |
|  | **I11**: Increasing patient satisfaction. Here quality goes before cost savings in my opinion. |  |
|  | **I12**: If you are able to organize processes smarter, that will always benefit the patient. |  |
|  | **Increased job satisfaction** |  |
| Increase job satisfaction | **I4**: Hospitals want to offer a safe work environment and they want employees to enjoy their work because that is only beneficial. |  |
|  | **I4**: If you do not have to check inventory anymore because that is automated, then people can offer greater added value and they have more pleasure in their work and do not have to perform that repetitive work anymore. |  |
|  | **I7**: Employees are still needed but the repetitive and boring tasks can be reduced so that we can also monitor that easier eventually. |  |
|  | **I12**: If we are talking about the most important results you can achieve, then I am talking about increasing job satisfaction of the healthcare workers and decreasing outflow. |  |
| Reduce manual labor | **I2**: A lot of things are being done manually with many people so many costs could be saved if that is not necessary anymore. |  |
|  | **I5**: We want to reduce manual labor because many tasks are being done manually which could be performed by a system. |  |
| Reduce workload | **I8**: You have to implement those kinds of automations, especially where the work is really heavy for employees. |  |
|  | **I9**: If you can make the logistics process less labor-intensive, you can at least save time when looking for solutions the moment something goes wrong. |  |
|  | **I12**: Healthcare workers have a high workload and work pressure. By digitalizing certain tasks, you can take away some tasks and that makes them happy. |  |
|  | **Operational efficiency** |  |
| Increase supply chain efficiency and effectiveness | **I1**: From the data, you can generate a stable data stream to organize your supply process better and more efficient. |  |
|  | **I4**: The more supplies you have, the more you have to manage since it needs to be purchased, coordinated and transported. |  |
|  | **I7**: If you look at the supply process, that could be more simple and effective since we often don’t know where our supplies are. |  |
|  | **I10**: You want to organize your tasks more efficient. That could be a benefit for us. |  |
| Minimize human error | **I3**: If the order is good, it is good. However, in reality, we order via e-mail or pdf. Then the supplier has to copy that data by typing it into their own system with all the mistakes that come with it. |  |
|  | **I6**: People forget to scan or do not see it well or someone grabs something in the meantime. So no, that could be done a lot better and IoT could play an important role. |  |
|  | **Operations automation** |  |
| Automate ordering process | **I1**: No one has to go to the inventory room anymore. The system knows when we are almost out of stock because the weight is low and then it will automatically send out an order. |  |
|  | **I3**: We are working on a pilot with smart warehouse racks. In the room, there are cameras which signal what you take out. They recognize the product based on a QR code and then the smart cameras register the usage and allow for an automatic order. |  |
| Automatic inventory management | **I2**: If you deliver the goods to the inventory room the system knows the inventory levels. If you then take something out, it also knows the new levels. |  |
|  | **I11**: You could equip all supplies with RFID tags so you can see the number of batteries that are in stock at the press of a button without needing an employee to come by. |  |
| Robots for logistic tasks | **I8**: Robots can be used to allow for automatic goods transport through our tunnels. |  |
|  | **I9**: We need to automate, digitalize, and robotize more to increase productivity. |  |
|  | **Regulatory requirements** |  |
| New laws and regulations as an enabler | **I7**: There is European legislation coming, I believe in 2025, which allows you to use more track and trace. | Strategy-related drivers  Strategy-related drivers (continued) |
|  | **I11**: There is more and more legislation and regulation which requires suppliers to provide more information with the delivery. |  |
|  | **Shift in focus** |  |
| Construction of new building taking into account possibilities for logistics | **I5**: They want to build new and with that it fits a new logistics concept. So that helps to change certain things. |  |
|  | **I6**: Then we say, if we are going to have to buy new access points soon, let’s look at what points can enable track and trace. In that way, you can keep investment costs minimal. |  |
| COVID-19 shifted focus to SCM | **I3**: Thanks to COVID-19 it has become apparent that without supplies we cannot provide care. So, it has become less obvious that the supplies are always there. |  |
|  | **I4**: You see a movement taking place. So, I think COVID-19 has done something good in healthcare. |  |
| Urgency to innovate is increasingly acknowledged | **I5**: We are behind 8 years, and everyone here knows it. So, that helps to increase the sense of urgency to change. |  |
|  | **I9**: IT and management start to slowly get convinced that we have to perform the same amount of work with fewer employees and that we thus need more technological support. |  |
|  | **I10**: Als a company we have to go along with it. They are slowly defrosting and start to see the possibilities. So, we are going in the right direction but we are not there yet completely. But slowly people start to see the benefits. |  |
|  | **Collaboration with suppliers** | Supply chain-related drivers  Supply chain-related drivers (continued) |
| Digitization as prerequisite for cooperation | **I1**: In order to work with us you need the data links. So the hospital must go along in the digitalization because otherwise, it cannot collaborate with us. |  |
| Information-sharing with suppliers | **I7**: Especially with COVID-19, some disruptions you do not detect immediately. There you do miss something. If the supplier would have known that the usage of a certain supply would rise fast, perhaps you could have done something to prevent the shortages. |  |
|  | **I8**: We miss out on information-sharing with suppliers. Contact with them is something we need to intensify. |  |
|  | **I9**: I think throughout the supply chain you want to receive information as early as possible from your suppliers about your orders that are coming. |  |
|  | **I10**: We believe that there is much more information available than what we get. However, that goes both ways, from our side to suppliers and from suppliers to us. |  |
|  | **I11**: We barely share any information, at least not digitally. That is also one of the problems. |  |
|  | **Increased traceability** |  |
| Ability to check circumstances of transport | **I3**: With the use of temperature sensors, which may be somewhat more expensive, you can check if pharmacy supplies for instance have been transported below -20 degrees at all times. |  |
|  | **I7**: If we have stored something in a sterile room, it can last longer than when we have it in a room with doors open. So the better the climate in the whole chain, the better you can indicate whether something is still good. That could save a lot of packaging as well eventually. |  |
| Tracing of medical supplies and devices | **I3**: Track and trace can be used so I know exactly where that container with bandages is located an ensure that no containers are lost. |  |
|  | **I8**: You want to know where your goods are and where it is now, not where it was before. So you can also ensure that goods are coming back. |  |
|  | **I12**: If you know where certain equipment is located, you can connect to see when that equipment needs to be inspected or when it needs maintenance. |  |
|  | **Increased transparency** |  |
| Better insight in arrival of supplies | **I3**: We would really like to know by means of GPS where a truck is and how long it will take to get here. |  |
|  | **I8**: We do not have a forecast. We do not know what will be delivered tomorrow. We do have agreements with some suppliers about when they will be here but we also do not know what they will deliver exactly. |  |
| Increased transparency throughout chain | **I5**: We have had some ideas but we want to get more transparency towards the suppliers. |  |
|  | **I5**: We want more insight into the supply chain, traceability up until the patient. |  |
