# Supplementary material for: Drivers and Barriers to Implementing the Internet of Things in the Health Care Supply Chain: Mixed Methods Multicase Study
Source: J Med Internet Res. 2023 Sep 20;25:e48730. doi: 10.2196/48730 (PMC10551782; doi:10.2196/48730)
Supplement: Multimedia Appendix 7 [file jmir_v25i1e48730_app7.docx]

**Multimedia Appendix 7.** Data structure barriers to implementing IoT in the healthcare supply chain

| **First-order concept** | **Second-order theme** | **Aggregated dimension** |
| --- | --- | --- |
|  | **Implementation costs are too high** | Financial barriers  Financial barriers (continued) |
| High costs | **I4**: There are track and trace solutions but these are still too costly so that break-even point cannot be achieved yet. |  |
|  | **I6**: I name it as a third but it could have better been the first barrier, that is the financials. |  |
|  | **I7**: I think it also depends on the application but we have had projects internally and then a cost estimate was made but eventually the costs were a lot higher because many physical adjustments would have to be made in the building. |  |
|  | **I8**: Over the years, a lot of money had to be saved and then you start to look where you can cut back, well on the supporting processes. But at a certain moment in time, you do not have the space to cut back further. Meanwhile, you do need some free space in order to be able to experiment and innovate. |  |
|  | **I11**: You will need to label every good with an RFID tag. For implants that is not a problem, as it already costs a couple of thousand euros, however, in the case of cotton swabs or towels, it becomes too expensive. |  |
| Fragmented budget structure | **I4**: Hospitals have many separate cost centers and separate budgets. There is a lack of an adequate integrated approach. |  |
|  | **I4**: The costs often end up with one department whilst the benefits can be seen throughout different departments or even throughout the whole chain. |  |
|  | **I12**: We look at processes within the hospital from an integrated view and we see that by investing in one place, benefits are achieved in multiple other departments. But that is often difficult for hospitals as one department has to pay whilst other departments get something in return. |  |
|  | **Trouble building a valid business case** |  |
| Difficult to monetize certain benefits | **I6**: There are several qualitative benefits from track and trace for instance that cannot directly be expressed in monetary value. |  |
| Uncertain whether benefits will compensate costs | **I5**: It is quite an administrative process because in the first phase there is already a lot of money needed and the whole process actually is quite costly. To get that money, it is not the case that we have a couple of million euros available just like that. |  |
|  | **I6**: It is difficult to get the financials. A business case? Never mind, too difficult. So, culture plays a role as well definitely. |  |
|  | **I11**: The question is whether we can establish a valid business case for such automation. |  |
|  | **Difficult to integrate into existing processes** |  |
| Difficult to standardize processes | **I11**: That is something we are struggling with a lot since we have no standardized processes. Doctor Janssen operates in a bit of a different way than doctor Pieters and as a result, may need different supplies for the same surgery. | Strategy-related barriers  Strategy-related barriers (continued)  Strategy-related barriers (continued) |
|  | **I11**: These are decisions that may affect the surgeon’s professionalism because that surgeon always works with that suture and not a different kind. |  |
| Large number of suppliers | **I1**: We would like to have GPS systems to locate orders coming in but it is more difficult to organize because we have way more suppliers. |  |
|  | **I11**: Because we are a university medical center, we provide care but also research and education and that is a really broad field. For providing care alone you already have many different specialisms and suppliers. However, because we also do research and education, I wouldn’t be able to think of another environment with this kind of diversity in its supplies and processes. |  |
|  | **I11**: Generally, we keep the door closed. Also because we have many different suppliers and I do not want to force my employees to keep track of inventory in four different systems. |  |
|  | **Lack of organization-wide coordination** |  |
| Lack of integrated approach | **I2**: There is no integrated view and realization that you can also improve care with techniques that are being implemented outside the core care process. |  |
|  | **I4**: I think it is a real obstacle that hospitals are really a group of different islands. They are eager to innovate but they are not always capable of organizing, also because they do not have enough knowledge and skills themselves. |  |
|  | **I7**: For that, we are too much on different islands, I think. And I hope that our strategy journey will enable more interconnectedness if it comes to working together and looking at the bigger picture. Not looking at each department by itself but as a bigger picture. |  |
|  | **I11**: Then you see that a challenge for us is the fact that our whole supply chain is organized as several distinct departments. |  |
| Short-term view | **I4**: Looking at how we are going to do things tomorrow. That is something they are not very good at. |  |
|  | **I5**: The healthcare system is completely built to solve a problem immediately as soon as it appears. |  |
|  | **I12**: In the healthcare industry, everything evolves around today. Often they are not thinking about tomorrow. |  |
| The larger the organization, the slower change can happen | **I1**: It is more that, the bigger the organization, the longer it takes to get something done. |  |
|  | **Lack of urgency to innovate supply chain processes** |  |
| Lack of attention for supporting processes | **I2**: There is a lot of attention for the primary healthcare process and everything outside of that serves as support and something that costs money. So it is seen as an extra. |  |
|  | **I3**: Of the total costs, 66% are staff expenses. Then, you have a couple million left. From that, a large part is fixed expenses like energy costs, building costs, investments, etc. Then, a small part from that is supply chain so it is just not on the radar. |  |
|  | **I7**: The new building looks very nice, but it could have been better designed to facilitate the supporting processes. Especially if you would think about the new technologies that are coming, you should keep that in mind. |  |
|  | **I8**: Only the necessary changes are implemented in order to sustain the current operations instead of looking to develop new opportunities. |  |
|  | **I12**: In the Netherlands, the healthcare industry is rather conservative and they are lacking behind in terms of digitalization. If they are looking at ways to digitalize, they are mainly looking at how to digitalize the primary process of providing care. |  |
| Supply chain management is not core business | **I3**: Supply chain management is just not our core business and you notice this in everything. |  |
|  | **I3**: The healthcare industry lags behind 10 years and especially in the area of supply chain management. |  |
|  | **I4**: Everything evolves around providing care. Providing care is seen as their purpose and as long as that goes well, they are getting paid. It is and stays a governmental institution in the end. |  |
|  | **I8**: The first thing that is important is the electronic health record. Everything evolves around that. |  |
|  | **Lack of cooperation with suppliers** |  |
| Suppliers unable to cooperate | **I1**: Sometimes a supplier does not have the data to cooperate. | Supply chain-related barriers |
| Suppliers unwilling to cooperate | **I1**: Sometimes a supplier says that they simply do not want to help. |  |
|  | **I5**: That idea died kind of because there were not enough suppliers who wanted to support it. They did not want to deliver the necessary data. |  |
|  | **I9**: The supplier does not always want to tell you that they have problems with their production process because they do not want you to seek an alternative supplier. |  |
|  | **I12**: The exchange of data is being blocked for a large part by large providers of electronic patient records. |  |
|  | **Technology immaturity** | Technology-related barriers  Technology-related barriers (continued) |
| Healthcare industry lags behind on data collection and analysis | **I5**: Data is really one of the biggest problems in hospitals, or for our hospital at least it is a problem to get the data in order. |  |
|  | **I11**: There have not really been initiatives to implement IoT. That is mainly due to the fact that we have only now started to work on the information side. |  |
| Infrastructure is not ready for technological innovations | **I2**: What you need is a network in your house with enough scan points in order to function well. But for instance, in the basement the internet coverage is low, so you have to install a lot of transmitters and receivers. |  |
|  | **I8**: I think an important precondition is that you are ahead with your ICT systems because you need those. I cannot say that we are very much ahead with our systems. In healthcare in general not but especially not in the logistics area. |  |
|  | **I9**: We have not gotten as far as we would have wanted to because there was no money to invest in new infrastructure, so we could not hang Bluetooth modules everywhere. But on the other side, the existing infrastructure is not at a sufficient level to allow for implementation. |  |
| Many more steps to take before IoT | **I5**: I believe that it is also a matter of the level of maturity. I can imagine that if you have already automated large parts of your process, it becomes easier to adopt new technologies. The different worlds lay closer to each other. I think that IoT has a maturity level six and as I said, we are really still on level zero. We are doing things manually that could be done by IoT but I think there are too many steps that should be taken first. |  |
|  | **I11**: We can still take a lot of steps and I think that if we would now implement IoT, the benefits we would reap from that are lower than the benefits of the steps that should be performed first. |  |
|  | **Security constraints** |  |
| Healthcare industry is highly regulated market | **I9**: We are in a highly regulated but also highly self-regulated market. So we cannot just say, let’s send all patients who have had a vascular surgery home after one day instead of three days. There are too many guidelines as well as standards of care to just change that. |  |
| Strict laws and regulations | **I6**: We have to obey those kinds of rules. The hospital is of course really afraid that if something goes wrong regarding privacy and the General Data Protection Regulation, we get high fines. And if something goes wrong with security and our systems go down, it will be printed in the papers that care delivery was jeopardized. |  |
|  | **I9**: If you look at newer equipment, you see that implementation within such a hospital infrastructure is quite tricky in many cases because the supplier demands access to your network which cannot be made available due to regulations. |  |
|  | **Afraid innovation will fail and cause problems** |  |
| Fear of failure due to earlier problems | **I5**: There have been large problems with the implementation of the ERP-system twice. This involved so many problems and asked a lot from the organization that it has created resistance. Therefore, these kinds of optimization opportunities did not really take off. | User-related barriers  User-related barriers (continued)  User-related barriers (continued) |
|  | **I7**: With every change that we execute here, people always look back and say: ‘But that time it didn’t go well’. |  |
|  | **I9**: You are talking about relatively high amounts of money for the healthcare industry and we do not have the space to gamble. If we make a wrong choice and lose half a million then we lose about 20% of the total profits. |  |
| Lack of initial trust in success of technological innovation | **I1**: The first time I heard it, I thought: ‘If it is a piece of paper and you start weighing, it will break’.  **I2**: Employees will react with mistrust until they experience that it actually works. |  |
|  |  |  |
|  | **Lack of knowledge and skills** |  |
| Healthcare managers lack business background | **I4**: It is really a current issue that one finds it difficult to organize the supporting processes well. You often see that healthcare managers try their best with good intentions. But many people in healthcare have a healthcare background and as a result, they have a different way of thinking than managers with a real business background. |  |
|  | **I6**: That is healthcare. And I know after 20 years in the healthcare industry that the people are gentle towards patients but also within management. Often these managers have advanced from healthcare positions. But as a result, they are also gentle towards suppliers and I come from the private sector and I believe we need to act more commercial at times and be clear in what we want and need from suppliers. |  |
| Unable to implement change | **I3**: Everybody does want to change but we are just not very good at it. |  |
|  | **I4**: I think that hospitals really want to but that they are not capable of organizing it themselves as the hospital consists of so many islands and because they do not have the knowledge and skills in-house. |  |
|  | **I5**: People want to do this but how to do it and how it will look eventually, that is difficult for everyone to grasp. So, to actually change and restructure the work is what people find difficult in my experience. |  |
|  | **I11**: You notice that the desire to change is present but the capability or the knowledge and skills are lacking to actually go through an organization-wide change and process change. |  |
| Unfamiliarity with possibilities | **I2**: People stay with what works now also because they are not familiar with what other things are also possible. |  |
|  | **I3**: I know what IoT is and I know what supply chain is but I do not see the link. |  |
|  | **I8**: A lack of knowledge is a reason why there have not been initiatives. Knowing what you could implement is really important I think. |  |
|  | **Resistance to change** |  |
| Conservative attitude | **I5**: That is often a nice picture. If you ask: ‘Who wants change?’, then everyone says yes. But if you ask: ‘Who wants to change?’, then everyone is reluctant. |  |
|  | **I10**: We sometimes joke about it but there are a lot of people here saying: ‘Why would we do that? It works well now’. But yes, things can be done better. |  |
|  | **I11**: That being said, especially if we look at the supporting processes but also in providing care, we are quite a conservative organization. |  |
| Cultural change is required | **I2**: It requires a cultural transformation within the organization.  **I4**: However, it is and remains a cultural change and that always goes along with some resistance in the beginning. |  |
|  |  |  |
|  | **I11**: The consequences of the implementation of IT on your work processes and the consequences of that on the professional identity of employees is something that we really struggle with. So, we see a lot of resistance to the introduction of these kinds of things. |  |
| Fear of being replaced | **I7**: If you tell them that they will have less work to do, they immediately ask whether they are then still necessary. |  |
|  | **I8**: The transport within the hospital is done by employees through the basement. You could do this with robots but the employees will not appreciate this as these robots will take over their work. |  |
|  | **I10**: Employees fear that they will be replaced. Employees within logistics generally have fewer qualifications, so they are not always open to these kinds of ideas. |  |
