# Supplementary material for: Drivers and Barriers to Implementing the Internet of Things in the Health Care Supply Chain: Mixed Methods Multicase Study
Source: J Med Internet Res. 2023 Sep 20;25:e48730. doi: 10.2196/48730 (PMC10551782; doi:10.2196/48730)
Supplement: Multimedia Appendix 8 [file jmir_v25i1e48730_app8.docx]

**Table S1.** Drivers identified by interviewees. SCM: supply chain management.

|  |  | Employees involved in executing projects | | | | | | IT employees | | | | SCM managers | |
| --- | --- | --- | --- | --- | --- | --- | --- | --- | --- | --- | --- | --- | --- |
| **Aggregate dimensions** | **Second-order themes** | **I1** | **I4** | **I5** | **I7** | **I10** | **I12** | **I2** | **I6** | **I9** | **I11** | **I3** | **I8** |
| Financial drivers | Cost savings | ✔ | ✔ | ✔ |  | ✔ | ✔ | ✔ | ✔ |  | ✔ |  | ✔ |
|  | Reduced healthcare expenses and insurance for patients |  | ✔ |  |  |  |  |  |  | ✔ |  |  | ✔ |
|  |  |  |  |  |  |  |  |  |  |  |  |  |  |
| Operational drivers | Creation of valuable info |  | ✔ |  | ✔ | ✔ | ✔ |  | ✔ | ✔ |  | ✔ |  |
|  | Higher agility | ✔ | ✔ |  |  |  |  | ✔ |  | ✔ | ✔ | ✔ |  |
|  | Higher employee productivity | ✔ | ✔ | ✔ | ✔ | ✔ | ✔ | ✔ |  | ✔ | ✔ | ✔ | ✔ |
|  | Improved inventory management | ✔ | ✔ | ✔ | ✔ | ✔ | ✔ | ✔ | ✔ | ✔ | ✔ | ✔ | ✔ |
|  | Improved quality of care | ✔ | ✔ |  | ✔ |  | ✔ |  | ✔ | ✔ | ✔ | ✔ |  |
|  | Increased job satisfaction | ✔ | ✔ | ✔ | ✔ | ✔ | ✔ | ✔ |  | ✔ | ✔ | ✔ | ✔ |
|  | Operational efficiency | ✔ | ✔ | ✔ | ✔ | ✔ | ✔ |  | ✔ | ✔ | ✔ | ✔ |  |
|  | Operations automation | ✔ | ✔ | ✔ | ✔ | ✔ | ✔ | ✔ | ✔ | ✔ | ✔ | ✔ | ✔ |
|  |  |  |  |  |  |  |  |  |  |  |  |  |  |
| Strategy-related drivers | Regulatory requirements |  |  |  | ✔ |  |  |  |  |  | ✔ |  |  |
|  | Shift in focus |  | ✔ | ✔ | ✔ | ✔ |  |  | ✔ | ✔ | ✔ | ✔ | ✔ |
|  |  |  |  |  |  |  |  |  |  |  |  |  |  |
| Supply-chain-related drivers | Collaboration with suppliers | ✔ |  |  | ✔ | ✔ |  |  |  | ✔ | ✔ | ✔ | ✔ |
|  | Increased traceability | ✔ | ✔ |  | ✔ | ✔ | ✔ | ✔ | ✔ | ✔ | ✔ | ✔ | ✔ |
|  | Increased transparency | ✔ |  | ✔ | ✔ |  |  |  |  | ✔ |  | ✔ | ✔ |

**Table S2**. Barriers identified by interviewees. SCM: supply chain management.

|  |  | Employees involved in executing projects | | | | | | IT employees | | | | SCM managers | |
| --- | --- | --- | --- | --- | --- | --- | --- | --- | --- | --- | --- | --- | --- |
| **Aggregate dimensions** | **Second-order themes** | **I1** | **I4** | **I5** | **I7** | **I10** | **I12** | **I2** | **I6** | **I9** | **I11** | **I3** | **I8** |
| Financial barriers | Implementation costs are too high | ✔ | ✔ | ✔ | ✔ | ✔ | ✔ |  | ✔ | ✔ | ✔ | ✔ | ✔ |
|  | Trouble building a valid business case |  | ✔ | ✔ | ✔ | ✔ |  | ✔ | ✔ | ✔ | ✔ |  | ✔ |
|  |  |  |  |  |  |  |  |  |  |  |  |  |  |
| Strategy-related barriers | Difficult to integrate into existing processes | ✔ |  |  |  |  |  |  |  | ✔ | ✔ |  | ✔ |
|  | Lack of organization-wide coordination | ✔ | ✔ | ✔ | ✔ | ✔ | ✔ | ✔ |  | ✔ | ✔ |  | ✔ |
|  | Lack of urgency to innovate supply chain processes | ✔ | ✔ |  | ✔ | ✔ | ✔ | ✔ | ✔ | ✔ | ✔ | ✔ | ✔ |
|  |  |  |  |  |  |  |  |  |  |  |  |  |  |
| Supply-chain-related barrier | Lack of cooperation with suppliers | ✔ |  | ✔ |  |  | ✔ |  |  | ✔ | ✔ |  |  |
|  |  |  |  |  |  |  |  |  |  |  |  |  |  |
| Technology-related barriers | Technology immaturity | ✔ |  | ✔ |  |  | ✔ | ✔ | ✔ | ✔ | ✔ | ✔ | ✔ |
|  | Security constraints |  |  |  |  |  | ✔ |  | ✔ | ✔ |  |  |  |
|  |  |  |  |  |  |  |  |  |  |  |  |  |  |
| User-related barriers | Afraid innovation will fail and cause problems | ✔ |  |  | ✔ | ✔ | ✔ | ✔ | ✔ | ✔ |  |  |  |
|  | Lack of knowledge and skills | ✔ | ✔ | ✔ |  | ✔ |  | ✔ | ✔ |  | ✔ | ✔ | ✔ |
|  | Resistance to change |  | ✔ | ✔ | ✔ | ✔ | ✔ | ✔ |  | ✔ | ✔ |  | ✔ |
